# Bone marrow mesenchymal stem cells regulate the dysfunction of NK cells via the T cell immunoglobulin and ITIM domain in patients with myelodysplastic syndromes

**DOI:** 10.1186/s12964-022-00985-2

**Published:** 2022-10-27

**Authors:** Zhaoyun Liu, Yixuan Guo, Lei Huang, Yue Jia, Hui Liu, Fengping Peng, Lixiang Duan, Hongkai Zhang, Rong Fu

**Affiliations:** 1 Department of Hematology, Tianjin Medical University General Hospital, Tianjin, People’s Republic of China; 2 Yuncheng Central Hospital, Yuncheng, Shanxi People’s Republic of China; 3 Shanghai Institute for Advanced Immunochemical Studies, ShanghaiTech University, Shanghai, 201210 People’s Republic of China; 4 State Key Laboratory of Medicinal Chemical Biology and College of Life Sciences, Nankai University, 94 Weijin Road, Tianjin, 300071 People’s Republic of China

**Keywords:** Myelodysplastic syndrome, Immune system, Natural killer cells, Bone marrow mesenchymal stem cells, T cell immunoglobulin and ITIM domain

## Abstract

**Background:**

Myelodysplastic syndrome (MDS) is a clonal disease of hematopoietic cells, characterized by hematopoietic cell hematopoiesis and a high risk of transformation into acute myeloid leukemia (AML). Although the underlying mechanism is unclear, MDS is often associated with immune system disorders, especially cellular immune abnormalities. We analyzed the number of lymphocyte subsets by flow cytometry assay and explored the alteration of lymphocyte subsets in MDS.

**Methods:**

Healthy controls, inpatients with primary MDS and patients with AML diagnosed from January 2017 to July 2021 were included. Flow cytometry assays were used to study lymphocyte subsets obtained from the bone marrow of the participants as well as changes in natural killer (NK) cell function. One-way analysis of variance and Student’s t-test were used to analyze the data.

**Results:**

We found a reduction in the number and function of NK cells in patients with MDS. By further measuring the activating and inhibitory receptors on the surface of NK cells, we found that the T cell immunoglobulin and ITIM domain (TIGIT) was the highest expressed marker on NK cells. Additionally, the expression of CD155, which is the ligand of TIGIT, was significantly higher than expressions of CD112 and CD113 on bone marrow mesenchymal stem cells (BMSCs).

**Conclusions:**

The co-culture results of BMSCs and NK cells demonstrated that BMSCs regulate NK cells through the TIGIT/CD155 interaction, indicating that NK cells play a vital role in MDS progression. BMSCs regulate the function of NK cells via TIGIT/CD155.

**Video Abstract**

**Supplementary Information:**

The online version contains supplementary material available at 10.1186/s12964-022-00985-2.

## Background

Myelodysplastic syndrome (MDS) is a type of malignant hematopoietic stem/progenitor cell clonal disease. Its main manifestations are different degrees of peripheral blood cell reduction, ineffective bone marrow and morbid hematopoiesis, and a high risk of progression to acute myeloid leukemia (AML) [1, 2]. The pathogenesis of MDS involves many factors, including molecular abnormalities and cytogenetic and immune disorders. Patients develop an imbalance of the immune surveillance system, with cellular immune deficiencies being the main cause [3, 4]; this mainly includes the depletion of T cells and natural killer (NK) cells. The hampered clearance against aberrant clones leads to immune escape and immune tolerance of malignant clonal cells. Hematopoietic suppressors secreted by various immune cells also promote excessive apoptosis of normal hematopoietic cells in the bone marrow [5–8]. Besides molecular genetic abnormalities of hematopoietic stem cells involved in the pathogenesis of MDS, functional exhaustion of immune cells is also essential.

In MDS, NK cell function appears abnormal, as previously described, and includes decreased antibody-dependent cytotoxicity and decreased direct NK cytolytic function. However, the biological mechanisms underlying these changes have not yet been determined [9–12]. NK cell activation is tightly regulated by a series of immune checkpoints [13]. NK cells express three main activating receptors: CD226 [14, 15], NK group 2D (NKG2D), and natural cytotoxicity receptor [13]. There are also some inhibitory receptors of NK cells, which are broadly divided into major histocompatibility complex class I (MHC class I) molecules (e.g., CD158a, CD158b, etc.) [16] and non-MHC class I inhibitory receptors (e.g., T cell immunoglobulin and ITIM domain [TIGIT], which was discovered in recent studies) [17]. TIGIT is a newly discovered co-stimulatory molecule with immunosuppressive effects and acts as an emerging inhibitory receptor shared by NK and T cells and a key inhibitor in the tumor immune cycle [18, 19]. TIGIT signaling inhibits NK cell cytotoxicity, granule polarization, and cytokine secretion through these pathways [20, 21]. TIGIT shares the common ligands CD155 and CD112 with CD226, CD226 can also bind to the adhesion protein CD113 [22–24]. TIGIT and CD226 competitively bind to CD155, and the affinity of TIGIT for CD155 is higher than that of CD226, thereby interrupting CD226-mediated activation and delivering inhibitory signals to T/NK cells [22].

Stromal cells, including bone marrow mesenchymal stem cells (BMSCs), which are in the bone marrow microenvironment of MDS, contribute to the altered bone marrow microenvironment, resulting in an abnormal hematopoietic microenvironment in patients with MDS [25]. BMSCs affect the expression and secretion of hematopoietic-related factors and participate in immune regulation and immune disorders in the tumor microenvironment [26]. BMSCs interact with NK cells in vitro [27, 28]. However, the potential reason for the immune system dysfunction is unclear. In this study, we collected the peripheral blood of patients with MDS to analyze the number of lymphocyte subsets by flow cytometry (FCM) assay and explore the alteration of lymphocyte subsets. We hypothesized that BMSCs regulate NK cells through the CD155/TIGIT pathway.

## Methods

### Ethics statements

This cohort study was conducted in accordance with the Declaration of Helsinki and was approved by the ethics committee of Tianjin Medical University General Hospital. (NO.IRB2021-WZ-180).

### Patient population and data collection

This study included 84 healthy controls (HCs), 130 inpatients with primary MDS, and 22 patients with AML initially diagnosed at the Department of Hematology, General Hospital of Tianjin Medical University, from January 2017 to July 2021. FCM and clinical data were collected. All patients with MDS were classified using the Revised International Prognostic Scoring System (IPSS-R) for disease risk. According to the IPSS-R classification, IPSS-R ≤ 3.5 and IPSS-R > 3.5 divided patients into the lower-risk MDS group and higher-risk MDS group, respectively (Table 1).

**Table 1:** Patients and clinical characteristics

Demographic variable	MDS(n = 130)
Age	
Median age, years(range)	63 (20–85)
Sex, n (%)	
Female	51 (39.23)
Male	79 (60.77)
IPSS risk score, n (%)	
Low	54 (41.54)
High	76 (58.46)
WHO 2016 classification, n (%)	
MDS del5q	5
MDS-U	0
MDS SLD	17
MDS RS SLD	18
MDS MLD	16
MDS RS MLD	7
MDS EB1	30
MDS EB2	37
Karyotype, n (%)	
Normal	101 (77.69)
Abnormal	29 (22.31)
Hb, median [g/L] (range)	75.5 (38–149)
Platelets, median [× 10^9^/L] (range)	67 (3–622)
Absolute neutrophil count, median [× 10^9^/L] (range)	1.62 (0.15–16.48)
Marrow blasts, median [%] (range)	2 (0–21)

### FCM

#### Lymphocyte surface receptors and functional assays

First, 100 μl of bone marrow (BM) samples were collected from MDS patients or HCs. Then, the bone marrow aspirations were stained with the following antibodies (BD Biosciences) at 4 °C for 15 min in the dark. Then 2 ml of lysing solution (BD Biosciences) was added to each tube to lyse erythrocytes for 10 min in the dark at room temperature, centrifuged at 1500 rpm for 5 min, and washed twice with phosphate-buffered saline (PBS). Finally, we analyzed the cells by FCM after washing the cells twice with phosphate-buffered saline (PBS). CytExpert Software (Beckman CytoFLEX) was used to analyze the FCM data.

The following antibodies (BD Biosciences) were used in this study: CD3-PerCP, CD56-APC-Cy7, TIGIT-FITC, CD226-APC, CD96-PE, NKG2D-PE-Cy7, CD107a-Bv421, IFN-γ-PE-Cy7, Perforin-Bv421, CD226-FITC, TIM-3-APC-Cy7, CD96-PE-Cy7, VISTA-PE-Cy7, PD-1- Bv421, LAG-3-Bv421, CD4-APC, CD8-PE, and CD56-PE. NK cells were labeled with CD3^−^CD56^+^, CD4^+^T cells with CD3^+^CD4^+^, and CD8^+^T cells with CD3^+^CD8^+^.

#### Mesenchymal stem cell surface receptor assay

When BMSCs were subcultured to third generation (P3) BMSCs, the cells in the logarithmic growth phase were obtained to observe the cell number and morphology under an inverted light microscope. After the cells were digested with 0.25% trypsin, the appropriate amount of cells was aspirated into a flow tube, washed once with PBS, and centrifuged at 1500 rpm for 5 min; the supernatant was discarded. BMSCs were labeled with CD105^+^, CD90^+^, CD73^+^, CD45^−^, and CD34^−^. CD155, CD112, and CD113 expressions were assessed by CytoFLEX flow cytometry (Beckman CytoFLEX); at least 5 × 10^5^ cells per sample were used for analysis, and the results were analyzed with FlowJo (V10).

Cells were labeled with antibodies (BD Biosciences), including CD90-FITC, CD73-Bv421, CD105-APC, CD34-PerCP, CD45-APC-Cy7, CD155-PE, CD112-PE, and CD113-PE, as per the manufacturer’s instructions.

#### Cell apoptosis

Cells were suspended in pre-cooled 1 × PBS and washed twice. Next, a 1 × binding buffer was added to resuspend the cells, which were then stained with a FITC Annexin V Apoptosis Detection Kit I (BD Biosciences) and incubated in the dark after mixing. Then, cells were analyzed within 1 h using CytoFLEX Software (Beckman CytoFLEX), and the percentages of apoptotic cells in all groups were compared.

### Cell culture

#### In vitro culture of BMSCs

BMSCs were cultured with Ficoll Hypaque density gradient centrifugation. Resuspended bone marrow mononuclear cells were placed into T25 flasks, the cell number was adjusted to 10^6^ cells/mL, and cells were incubated at 37 °C and 5% carbon dioxide. The culture medium for BMSCs comprised Dulbecco's modified eagle medium (DMEM) with 15% fetal bovine serum (FBS) (Gibco), 100 U/mL of penicillin and streptomycin (Solarbio) in DMEM/F-12 complete medium (Gibco). BMSCs were incubated in the culture medium for 72 h, followed by a half volume change to remove non-adherent cells, after which the medium was changed every 3–4 days until the appearance of adherent cells arranged in a fusiform radial or swirling pattern and the medium change was completed. When the cell confluence reached 80–90%, the cells were digested with 0.25% trypsin (Gibco) and passaged. P3 and fourth generation (P4) BMSCs were used in the follow-up experiments.

#### Sorting NK cells

We placed 5 mL of bone marrow blood from patients with MDS in a test tube containing heparin, and mononuclear cells were separated by density gradient centrifugation with a lymphocyte separation solution and counted with a cytometer under a microscope. We added the corresponding volume of magnetic beads (Miltenyi Biotec) in the tube containing the NK cell subgroup according to the number of cells labeled with antibody. After incubating the cells in the dark, they were sorted and collected under magnetic conditions. FCM was used to detect the sorting purity; we used 100 U/mL of interleukin (IL)-2, 10% FBS (Gibco), 100 U/mL of penicillin and streptomycin (Solarbio), and RPMI1640 medium (Gibco) for in vitro expansion culture.

#### Co-culture of BMSCs and NK cells

The BMSCs and NK cells from patients with MDS were co-cultured at a ratio of 4:1 and grouped according to the addition of different immune checkpoint inhibitors, including anti-TIGIT mAb (R&D Systems) and the CD226 agonist (WO 2020/023312 A1, Additional file 1: Fig. S1). After 6 days of incubation, FCM was used to detect the changes in NK cell function before and after co-cultivation. At the same time, the MDS cell line (SKM-1) was also co-cultured with NK cells at the effector cell: target cell ratios(E:T ratio) of 1:1, 5:1, and 10:1 for 6 h after co-cultivation using the Annexin V Cell Apoptosis Analysis Kit (BD Biosciences).

### Statistical analysis

All data are expressed as the mean ± standard error of the mean (normally distributed data) or median (25% percentile to 75% percentile) (non-normally distributed data). Data from three or more groups were compared using a one-way analysis of variance. Statistical difference between the two groups was analyzed using the Student’s t-test. Data were analyzed using Prism statistical software (version 7.00; GraphPad Software, Inc.). A *P*-value < 0.05 was considered statistically significant.

## Results

### The decrease of NK cells may be related to the prognosis of patients with MDS

Table 1 shows patients’ baseline characteristics. There were no differences in the sex ratio or age between patients with MDS and HCs (*P* > 0.05).

**Fig. 1:**
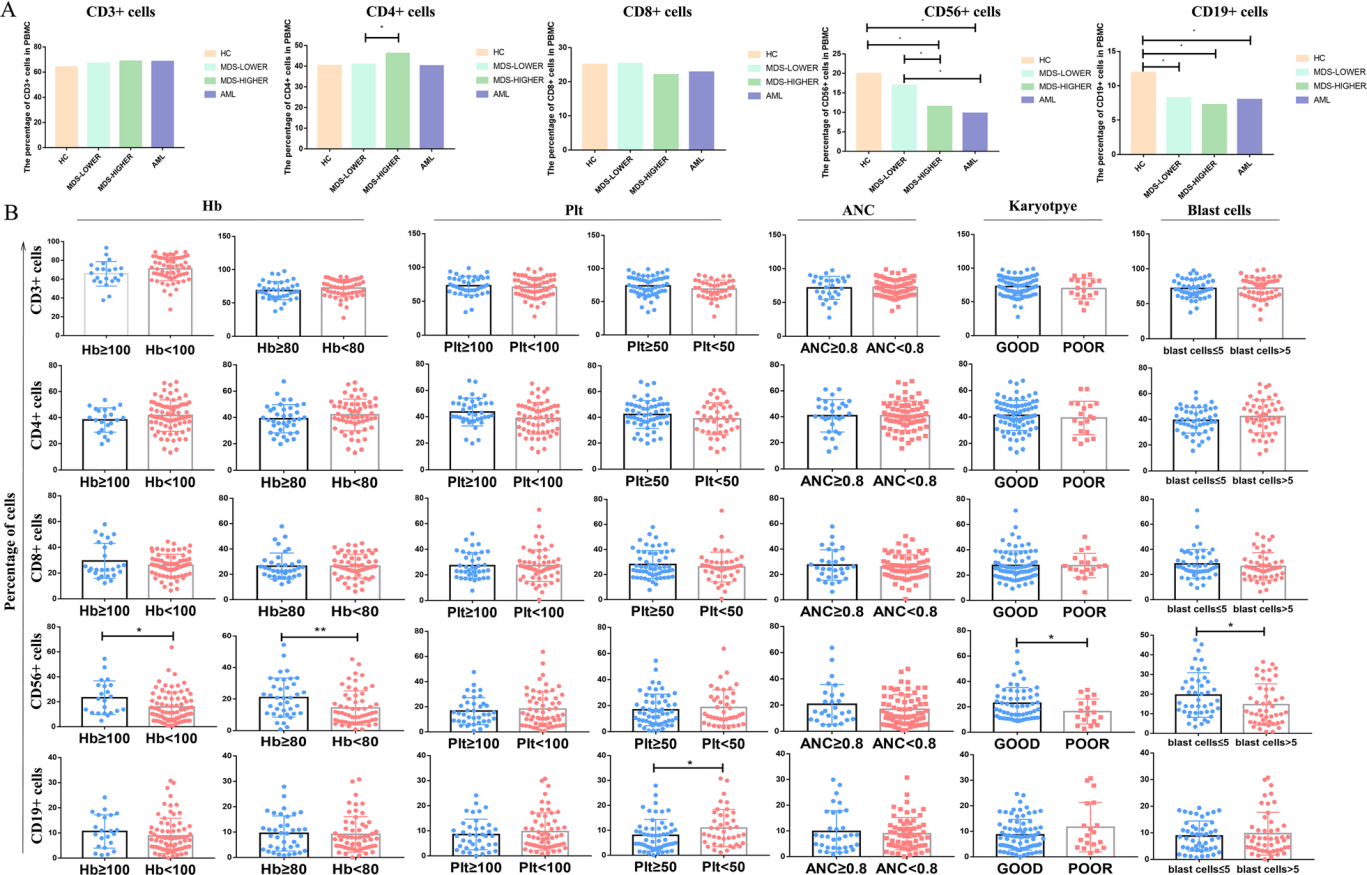
Decrease of NK cells may be related to prognosis of patients with MDS. **A**. The percentages of lymphocyte levels in healthy controls, patients with MDS, and patients with acute myeloid leukemia. **B**. The proportions of lymphocytes and NK cells according to Revised International Prognostic Scoring System staging-related factors, including Hb (less/more than 100 g/L or less/more than 80 g/L); Plt (less/more than 150 g/L or less/more than 50 g/L); ANC (less/more than 0.8 × 10^9^ L); Karyotypes (good or poor karyotypes); Blast cells (less/more than 5%). NK, natural killer; MDS, myelodysplastic syndrome; Hb, hemoglobin; Plt, platelets; ANC, absolute neutrophil count

We found that the percentage of NK cells in the HC group (19.94, 95% confidence interval [CI] 17.86–22.02, *P* < 0.001) was significantly higher than that in the MDS and AML groups. The percentage of NK cells in the higher-risk group of MDS (11.51, 95% CI 9.34–13.68) was significantly lower than that in the lower-risk group (16.91, 95% CI 13.85–19.97, *P =* 0.006), and the percentage of the higher-risk group was close to that of the AML group (9.77, 95% CI 6.33–13.20, Fig. 1A, Additional file 1: Table S1). The serum level of interferon gamma (IFN-γ), which is secreted mainly by NK cells, was also the highest in the HC group (5.50, 95% CI 3.74–7.26), followed by that in the MDS group and the AML group (2.64, 95% CI 1.91–3.37). Moreover, the higher-risk MDS group (3.26, 95% CI 2.45–4.07) had a significantly lower IFN-γ level than the lower-risk MDS group (4.80, 95% CI 3.66–5.95, Additional file 1: Table S2). CD8^+^ T cells and CD19 + cells had similar results as NK cells, but there was no significant difference between CD3^+^ cells and CD4^+^ T cells. To further investigate the relationship between NK cells and clinical characteristics, we compared the percentages of different immune cells among MDS-related clinical characteristics; significantly higher NK cell percentages were found in patients with a hemoglobin level ≥ 100 × 10^12^/L compared to those with a lower hemoglobin level (23.23, 95% CI 17.26–29.21 versus 15.77, 95% CI 13.00–18.55, *P =* 0.010). The levels of NK cells were higher in patients with blast cells ≤ 5% than in those with blast cells > 5% (19.51, 95% CI 16.09–22.93 versus 14.65, 95% CI 11.55–17.74, *P =* 0.027). Patients with better karyotypes also had higher NK levels than those with poorer karyotypes (22.75, 95% CI 19.47–22.03 and 16.26, 95% CI 11.36–21.15, *P =* 0.060; Fig. 1B (a–e)). No significant difference was found between NK cell levels with regard to platelet and neutrophil counts. CD8^+^ T cells behaved similarly to NK cells. Therefore, we found that the number of NK cells in patients with MDS decreased, which may be related to the prognosis of MDS.

### The function of NK cells from patients with MDS was decreased with the high expression of TIGIT

**Fig. 2:**
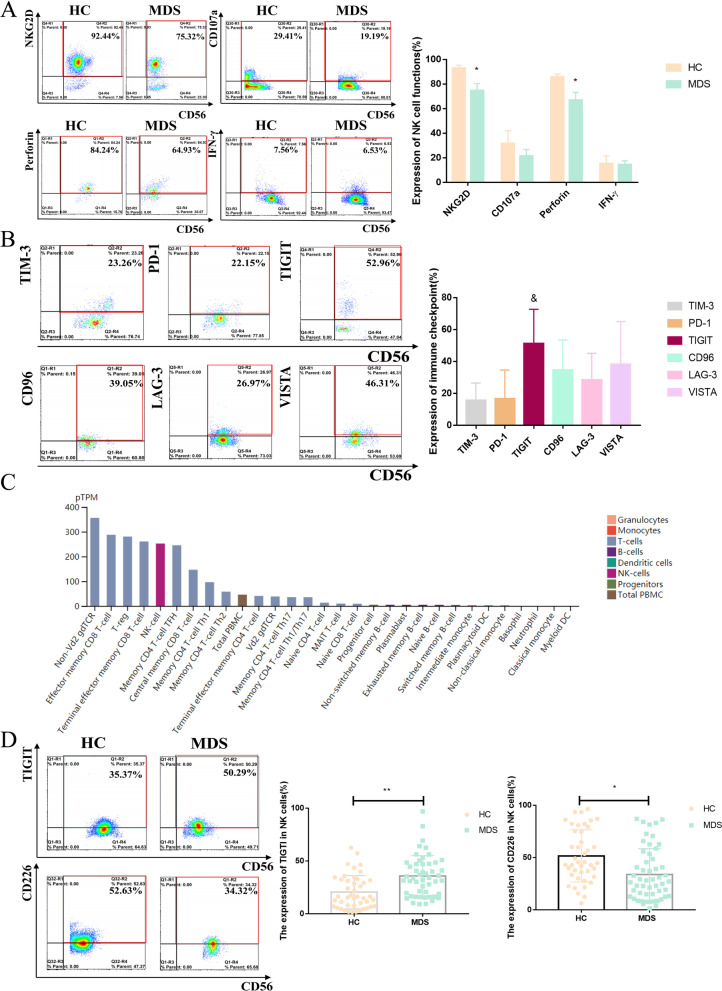
NK cell function in patients with MDS is decreased when TIGIT expression is increased. **A**. The expression of NKG2D, Perforrin, CD107a and IFN-γ in MDS patients were decreased compared to those in HCs. **B**. The expression of inhibitory receptors TIM-3, PD-1, TIGIT, CD96, LAG-3 and VISTA of NK cells in MDS patients. **C**. TIGIT RNA expression in normal human tissue plotted as RPKM. Data were obtained from the Human Protein Atlas Dataset available from proteinatlas.org. **D**. The expression of TIGIT was significantly increased on NK cells in MDS patients compared to that in HCs, whereas CD226 expression was decreased. The dot plots are the statistics of the expression of TIGIT and CD226, including patients with HCs (n = 44) and MDS patients (n = 50).* indicates a significant difference compared with the HC group, *P* < 0.05. ** indicates a significant difference compared with the HC group, *P* < 0.001. NK, natural killer; MDS, myelodysplastic syndrome; TIGIT, T cell immunoglobulin and ITIM domain; HCs, healthy controls; RPKM, rates per kilobase million

We found that the number of NK cells was decreased in patients with MDS. However, the function of NK cells with MDS patients is not clear. Therefore, we detected the activating receptors (NKG2D), a marker for degranulation (CD107a), cytotoxic granules (Perforin), and cytokine (IFN- γ) on NK cells from HCs and MDS patients using flow cytometry. The expression of NKG2D and perforin decreased significantly in MDS patients (Fig. 2A). To explore the reasons for the reduced number and function of NK cells in patients with MDS, we examined the expression of inhibitory receptors in NK cells. Our results showed that the expression of TIGIT on NK cells was the highest compared to that of TIM-3, PD-1, CD96, LAG-3, or VISTA (Fig. 2B). Moreover, it is known from protenatlas.org that TIGIT expression on the surface of NK cells is high compared with other immune cell types (Fig. 2C). Then, we detected CD226 and TIGIT expressions on the surface of NK cells from HCs and MDS patients. The expression of TIGIT on NK cells was significantly increased in the MDS group (35.69% ± 2.726%) compared to that in the HC group (20.35% ± 2.384%) (**P < 0.001). While the expression of the active receptor CD226 was lower in the MDS group (33.64% ± 3.408%) than in the HCs group (51.51% ± 3.848%) (*P < 0.05)(Fig. 2D). Moreover, we detected TIGIT and CD226 on CD8^+^ and CD4^+^ cells; no significant difference was found between the levels of TIGIT and CD226 on CD8^+^ and CD4^+^ cells from the MDS and HC groups (Additional file 1: Fig. S1), suggesting that the dysfunction of NK cells in patients with MDS may be related to the decreased expression of CD226 and the increased expression of TIGIT.

### BMSCs regulated NK cell function through the TIGIT/CD155

**Fig. 3:**
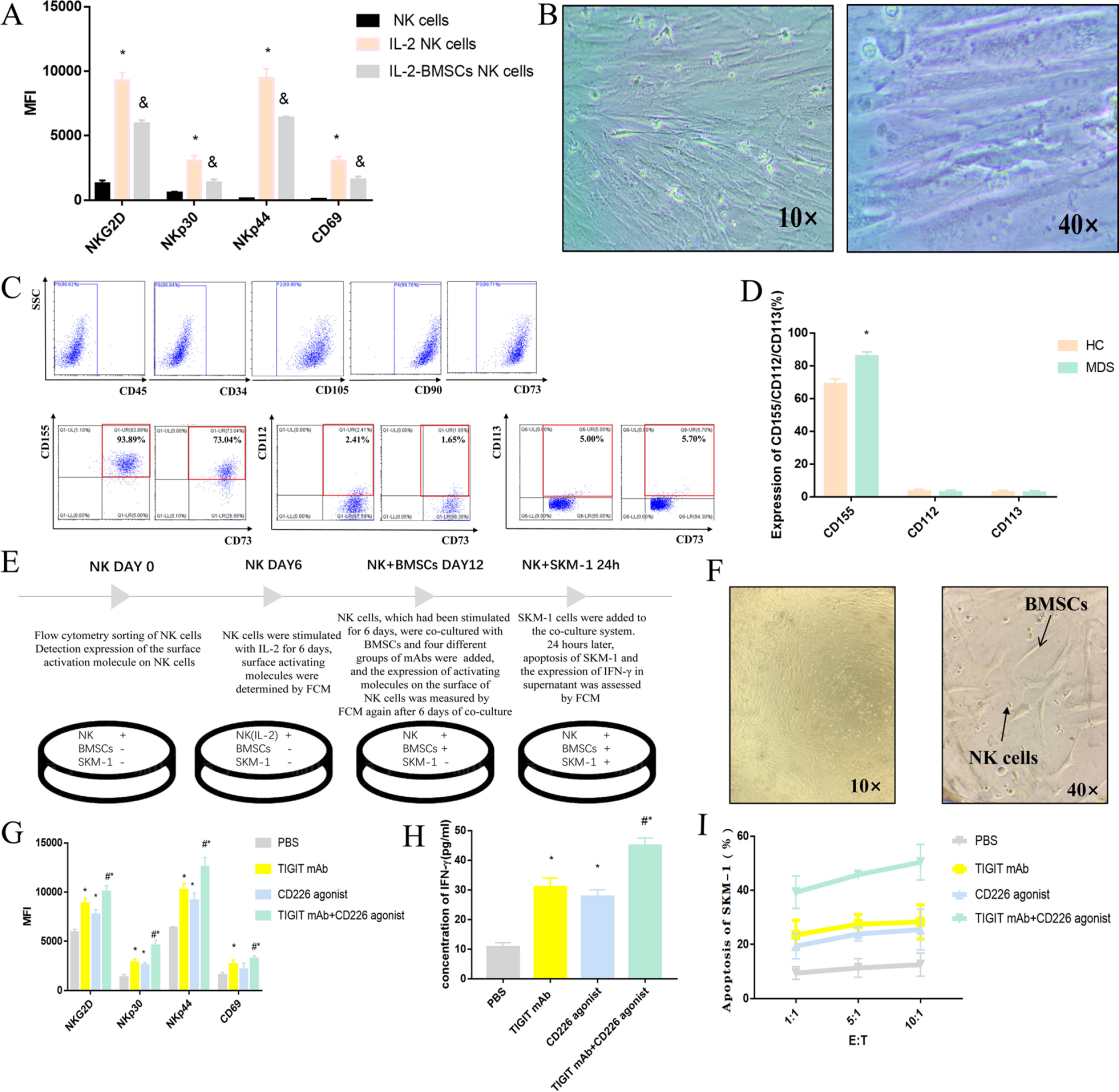
BMSCs regulated NK cell function through the TIGIT/CD155 pathway. **A**. NK cells were tested in freshly isolated cells, after 6-day cultured in 100 U/mL IL-2 and after 6-day co-cultured with BMSCs for the expression of NK cell surface activating receptors. *indicates a significant difference compared with the NK cell group, *P* < 0.05. & indicates a significant difference compared with the IL-2 NK cell group, *P* < 0.05. **B**. The morphology of BMSCs under the microscope (scale bar: 100 μm). **C**. Expression of typical markers with CD73, CD90, CD105, CD34, and CD45 of BMSCs. All the BMSCs used in subsequent experiments were third‐generation. **D**. Flow cytometry detection of CD155/CD112/CD113 expression levels on the surface of BMSCs. The expression of CD155 on BMSCs of MDS patients was significantly increased, but CD112 and CD113 were expressed at very low levels. **E**. Experimental procedure of co-culture of BMSCs and NK cells **F**. The morphology of the BMSCs-NK co-culture system under the microscope (scale bar: 100 μm). **G**. Expression of activating receptors NKG2D, NKp30, NKp44, and CD69 was measured by flow cytometry after blocking TIGIT and CD226 mAb. Data were expressed as MFI. **H**. Cytokine levels secreted by NK cells after blocking TIGIT and CD226 mAb. **I**. Results of cytotoxicity of NK cells in which NK cells were used as targets while SKM-1 cells were used as effectors. The apoptosis of SKM-1 cells gradually increased after the addition of TIGIT mAb and CD226 mAb. Data were expressed as a percentage of lysis. *indicates a significant difference compared with the PBS group. #indicates a significant difference compared with the TIGIT mAb and CD226 agonist groups, *P* < 0.05. BMSCs, bone marrow mesenchymal stem cells; NK, natural killer; TIGIT, T cell immunoglobulin and ITIM domain; MFI, mean fluorescence intensity; PBS, phosphate-buffered saline

It is known that BMSCs significantly inhibit T cell proliferation. Therefore, to evaluate whether BMSCs have the same inhibitory effect on NK cells, we examined the activating receptors expressed in freshly isolated NK cells (NK cells) and in NK cells that had been stimulated with 100 U/mL IL-2 for 6 days (IL-2 NK cells) and in the presence of BMSCs after stimulated with IL-2 for 6 days (BMSCs-IL-2 NK cells). After being stimulated with IL-2, NK cells exhibited upregulated expressions of NKp30 and NKG2D. Furthermore, IL-2 stimulated the expression of the NKp44 receptor and CD69, which is not expressed in fresh NK cells (Fig. 3A). When NK cells were cultured with BMSCs, the expressions of NKp30, NKG2D, NKp44, and CD69 were downregulated compared to IL-2–activated NK cells (Fig. 3A). These results illustrated that NK cells interacted with BMSCs. Next, we examined the expression of ligands recognized by NK cell receptors on the surface of BMSCs and found a significantly high expression of CD155 and low expression of CD112 and CD113 on the surface of BMSCs from MDS patients (68.74% ± 3.270% vs. 85.62% ± 2.835%)(*P < 0.05)(Fig. 3D). This finding suggests that BMSCs may regulate NK cell function through the CD155/TIGIT.

### Blocking TIGIT and activating CD226 could reverse the inhibitory effect of BMSCs on NK cells

To investigate the mechanism underlying the inhibitory effect of BMSCs on NK cells, we studied the role of channel receptors that may be involved in this phenomenon. We performed co-culture experiments with or without monoclonal antibodies (mAbs) against these receptors, including TIGIT and CD226. NK cells were co-cultured with BMSCs following IL-2 stimulation for 6 days. TIGIT mAb and CD226 agonist were added to the wells of NK-MSC co-cultures (see Fig. 3E, F). We also used these two reagents in combination to evaluate their possible superimposed effects. After 6 days, we detected the expression of activation molecules on the NK cell surface, the ability of NK cells to kill target cells and the ability of secreting cytokines. In the co-culture system, the expression of activating receptors NKG2D, NKP30, NKP44, and CD69 with TIGIT mAb and CD226 agonist were restored compared to the control group (*P < 0.05) (Fig. 3G). There was also a significant recovery in the secretion of IFN-γ with these two reagents (Fig. 3H). The expression of these activating receptors and secretion of IFN-γ increased significantly when the two reagents were used simultaneously compared to monotherapy groups (*P < 0.05). To examine the ability of NK cells to kill target cells, we added SKM-1 cells to the co-culture system. Apoptosis in SKM-1 cells was significantly higher in the groups with TIGIT mAb (27.47% ± 3.60%) and CD226 mAb (23.85% ± 2.55%) than that in the control group (11.34% ± 3.35%) (E:T ratio: 5:1). Furthermore, apoptosis in SKM-1 cells in the two agents (TIGIT and CD228 mAbs) combination group (45.78% ± 6.57%, E:T ratio) was significantly higher than that in the monotherapy groups (Fig. 3I). We found that the addition of TIGIT mAb and CD226 agonist counteracted the inhibitory effect exerted by BMSCs. And the two-agent combination group had a better effect in restoring NK cell function than in the monotherapy groups.

## Discussion

MDS includes cytogenetic and molecular abnormalities and cellular immune disorders [29]. In this disease, dysregulation of the immune system (including NK cells, abnormal cytokine milieu in T cells, and altered inflammation) may be a major pathophysiological abnormality of MDS [30]. Our research found that, besides fibroblasts, endothelial cells, and some tumor cells [31], CD155 is also highly expressed on BMSCs, and we demonstrated that BMSCs might directly inhibit NK cell function through the TIGIT/CD155 pathway, leading to NK cell depletion. To verify our hypothesis that BMSCs regulate NK cells through the CD155/TIGIT pathway, we used TIGIT mAbs in the co-culture system. First, we found that the TIGIT mAb group significantly enhanced the function of NK cells. The combination therapy of the TIGIT mAb and CD226 agonist further activated NK cells and produced a greater anti-tumor response. These results indicated that TIGIT mAb and CD226 agonist could restore NK cell function. Hence, it is reasonable to speculate that TIGIT and CD226 are both involved in the BMSC-mediated inhibition of NK cell function, and they seem to have a synergistic effect. We conclude that blocking TIGIT can enhance NK cell function and simultaneously upregulate CD226, achieving optimal clinical outcomes. Our research shows, for the first time, that the TIGIT blockade can reverse defective NK cell function in patients with MDS, consistent with previous reports in colon and breast cancers [32, 33].

Our research showed that the number of NK cells in patients with MDS was markedly reduced compared with that in HCs, while CD3^+^/CD4^+^/CD8^+^/CD19^+^ lymphocytes were not significantly different between these two groups. This finding is consistent with previously reported results [34, 35]. Our study results showed that the functional defection of NK cells was related to higher IPSS-R scores (IPSS-R > 3.5) in patients with MDS and that excessive blasts are also related to defective NK function. We also examined cytokine expression in the peripheral blood of patients with MDS and found that IFN-γ (Additional file 1: Table S2), which is predominantly secreted by NK cells, was significantly lower in patients with MDS than in HCs, which also suggested that a functional abnormality is present in patients with MDS. This result also validated that evasion of NK immune surveillance noted in previous studies may have important implications for MDS progression [35]. Therefore, we speculate that cellular immune abnormalities in MDS are dominated by abnormalities in NK cell number and function.

NK cells are innate lymphoid cells, and the activation of NK cells depends on the balance between cellular signals mediated by activating and inhibitory receptors [36]. As previously reported, the activating receptors include CD226 and NKG2D, and the inhibitory receptors include TIGIT, TIM-3, CD96, PD-1, VISTA, and LAG-3 [36–38]. We examined the expression of inhibitory receptors on NK cells, CD4^+^ T cells, and CD8^+^ T cells (Additional file 1: Fig. S1). Among these receptors, TIGIT, TIM-3, CD96, LAG-3, and PD-1 were abnormally expressed in myeloid-derived suppressor cells. Our results showed that TIGIT expression on NK cells was significantly increased (*P <* 0.05), whereas the immune checkpoints on the surface of CD4^+^/CD8^+^ T cells were not significantly different (*P* > 0.05). We also detected CD226 expression in CD4^+^ and CD8^+^ T cells and NK cells. The results showed that in patients with MDS, the expression of CD226 on the surface of NK cells decreased significantly, suggesting that the TIGIT/CD226 pathway may be a major contributor to abnormal NK cell immunity.

**Fig. 4:**
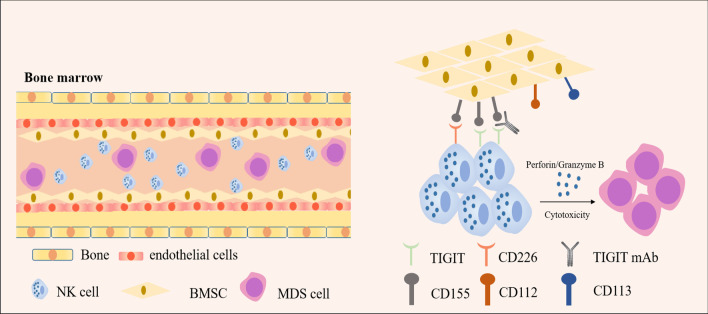
BMSCs regulated NK cell function through the TIGIT/CD155 pathway. BMSCs, bone marrow mesenchymal stem cells; NK, natural killer; TIGIT, T cell immunoglobulin and ITIM domain

An increasing number of studies have shown that alterations in the bone marrow microenvironment have a significant effect on the development and progression of MDS [39, 40]. BMSCs are important constituents in the hematopoietic stem cell niche, which can promote the formation of MDS and disease progression in terms of genetic, epigenetic, and molecular abnormalities [40, 41]. At present, research on the immune function of BMSCs in MDS is relatively limited. However, it is still clearly proven that the immune regulatory function of BMSCs promotes the progression of MDS and the disease transformation to AML [42]. BMSCs and NK cells can interact with each other in vitro [27]. Therefore, we co-cultured NK cells and BMSCs in vitro to investigate whether the function of NK cells changed. Our results showed that IL-2-stimulated NK cell surface activation molecules were significantly upregulated after 6 days. After the addition of BMSCs, BMSCs exhibited an inhibitory effect on NKp30 and NKG2D expression, which illustrated that BMSCs significantly inhibited NK-mediated cytotoxic activity. This study proved that BMSCs can block IL-2-induced activation of NK cell function. Moreover, we detected the expressions of CD155, CD112, and CD113, the ligands for TIGIT, on the surface of BMSCs and observed that the expression of CD155 was apparently higher in patients with MDS than in HCs. These results suggest that BMSCs may regulate the function of NK cells through the CD155/TIGIT pathway (Fig. 4).

## Conclusions

In conclusion, our research demonstrates that the CD155 /TIGIT pathway plays an important role in the regulation of NK cells by BMSCs. The inhibitory effect of BMSCs on NK cell function can be achieved by blocking TIGIT in NK cell-based immunotherapy in patients with MDS. However, because of the lack of animal validation, no direct evidence has been provided to support the use of the TIGIT blockade and CD226 activation in vivo in patients with MDS. Future studies should focus on demonstrating the in vivo efficacy of such treatments before their application in the clinical setting.

## Supplementary Information

**Additional file 1:** Supplementary tables and figures.

## Data Availability

All datasets that the conclusions of the paper rely on are available to readers and deposited in publicly available repositories.

## Abbreviations

MDS: Myelodysplastic syndrome
AML: Acute myeloid leukemia
NK: Natural killer
NKG2D: Natural killer group 2D
MHC class I: Major histocompatibility complex class I
TIGIT: T cell immunoglobulin and ITIM domain
BMSCs: Bone marrow mesenchymal stem cells
FCM: Flow cytometry
HCs: Healthy controls
IPSS-R: Revised international prognostic scoring system
PBS: Phosphate-buffered saline
P3: Third generation
DMEM: Dulbecco's modified eagle medium
FBS: Fetal bovine serum
P4: Fourth generation
CI: Confidence interval
IFN-γ: Interferon gamma
mAbs: Monoclonal antibodies
E:T ratio: Effector cell: target cell ratio
