# Supplementary material for: Bone marrow mesenchymal stem cells regulate the dysfunction of NK cells via the T cell immunoglobulin and ITIM domain in patients with myelodysplastic syndromes
Source: Cell Commun Signal. 2022 Oct 27;20:169. doi: 10.1186/s12964-022-00985-2 (PMC9615182; doi:10.1186/s12964-022-00985-2)
Supplement: Supplementary file 2 — Additional file 1: Supplementary tables and figures. [file 12964_2022_985_MOESM2_ESM.docx]

| immune cells | HC  N1=83 | MDS-LOWER  N2 =34 | MDS-HIGHER  N3 =50 | AML  N4 =22 | P | immune cells comparisons | | | | | |
| --- | --- | --- | --- | --- | --- | --- | --- | --- | --- | --- | --- |
|  |  |  |  |  |  | N1vsN2 | N1vsN3 | N1vsN4 | N2vsN3 | N2vsN4 | N3vsN4 |
| CD3+(%) | 63.88 (63.72,68.04) | 67.00 (65.32,72.68) | 68.61 (64.79,72.43) | 68.34 (63.03,73.63) | 0.282 | 0.161 | 0.090 | 0.366 | 0.949 | 0.725 | 0.669 |
| CD4+(%) | 40.22 (37.96,42.48) | 40.89 (38.39,43.38) | 46.06 (43.24,48.89) | 40.10 (35.57,44.63) | **0.006** | 0.548 | **0.002** | 0.735 | **0.014** | 0.492 | **0.014** |
| CD8+(%) | 25.06 (23.25,26.88) | 25.30 (22.36,28.23) | 22.06 (20.14,23.97) | 22.82 (18.61,27.03) | 0.305 | 0.537 | 0.134 | 0.659 | 0.080 | 0.456 | 0.517 |
| CD56+(%) | 19.94 (17.86,22.02) | 16.91 (13.85,19,97) | 11.51 (9.34,13.68) | 9.77 (6.33,13.20) | **<0.001** | 0.148 | **<0.001** | **<0.001** | **0.006** | **0.004** | 0.379 |
| CD19+(%) | 11.90 (10.86,12.94) | 8.19 (6.27,10.11) | 7.22 (5.98,8,45) | 7.97 (5.52,10.42) | **<0.001** | **0.001** | **<0.001** | **0.002** | 0.637 | 0.893 | 0.760 |

**Supplement data**

| Cytokines | HC  N1=33 | MDS-LOWER  N2=7 | MDS-HIGHER  N3=17 | AML  N4 =11 | P | Cytokines comparisons | | | | | |
| --- | --- | --- | --- | --- | --- | --- | --- | --- | --- | --- | --- |
|  |  |  |  |  |  | N1vsN2 | N1vsN3 | N1vsN4 | N2vsN3 | N2vsN4 | N3vsN4 |
| IL-2 | 4.93 (3.92,5.14) | 2.21 (0.43,3.98) | 3.19 (2.59,3.79) | 2.80 (2.33,3.26) | **<0.001** | **0.004** | **0.012** | **<0.001** | 0.193 | 0.497 | 0.155 |
| IL-4 | 3.53 (2.68,4.39) | 4.12 (2.40,5.84) | 5.31 (4.08,6.55) | 5.67 (4.19,7.15) | 0.055 | 0.533 | **0.050** | **0.017** | 0.253 | 0.258 | 0.689 |
| IL-6 | 4.84 (4.00,5.68) | 6.09 (2.05,10.13) | 7.58 (4.87,10.29) | 17.05 (7.16,26.94) | **0.001** | 0.565 | 0.180 | **<0.001** | 0,432 | **0.048** | **0.014** |
| IL-10 | 4.38 (3.66,5.09) | 5.20 (2.18,8,21) | 6.33 (4.40,8.25) | 7.75 (5.69,9.80) | **0.007** | 0.762 | 0.055 | **0.001** | 0.397 | 0.094 | 0.080 |
| IFN-Ƴ | 5.50 (3.74,7.26) | 4.80 (3.66,5.95) | 3.26 (2.45,4.07) | 2.64 (1.91,3.37) | **0.005** | 0.805 | **0.025** | **0.005** | **0.026** | **0.017** | 0.242 |
| TNF-α | 3.85 (3.28,4.41) | 3.11 (2.09,4.12) | 2.85 (2.15,3.56) | 2.24 (1.63,2.85) | **0.010** | 0.313 | 0.091 | **0.001** | 0.977 | 0.116 | 0.180 |

Table 1 Percentage of lymphocytes levels in healthy controls, MDS patients, and AML patients

Table 2 Secreted cytokine levels in healthy controls, MDS patients, and AML patients


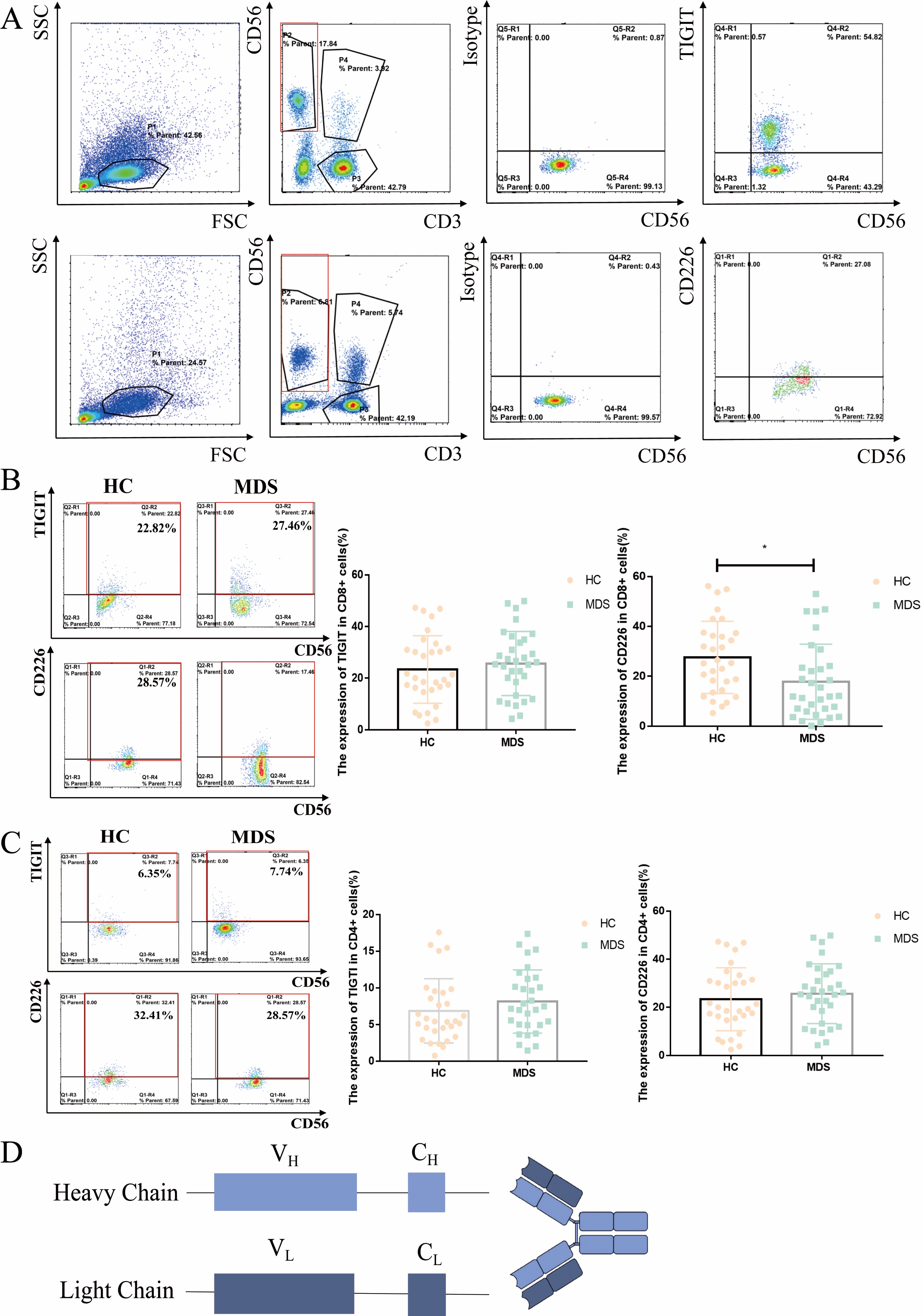


Figure1 A. NK cells were stained with CD3, CD56 flow antibodies. B. The expression of TIGIT and CD226 on the surface of CD8+ cells. C. The expression of TIGIT and CD226 on the surface of CD4+ cells. D.Schematic diagram of CD226 agonist.
